# The Ongoing Epidemics of Seasonal Influenza A(H3N2) in Hangzhou, China, and Its Viral Genetic Diversity

**DOI:** 10.3390/v17040526

**Published:** 2025-04-04

**Authors:** Xueling Zheng, Feifei Cao, Yue Yu, Xinfen Yu, Yinyan Zhou, Shi Cheng, Xiaofeng Qiu, Lijiao Ao, Xuhui Yang, Zhou Sun, Jun Li

**Affiliations:** 1 Hangzhou Center for Disease Control and Prevention (Hangzhou Health Supervision Institution), Hangzhou 310002, China; 2 Zhejiang Key Laboratory of Multi-Omics in Infection and Immunity, Hangzhou 310002, China

**Keywords:** influenza A/H3N2, whole-genome sequencing, phylogenetic analysis, Shannon entropy, antiviral resistance, Bayesian molecular clock, selective pressure, Pepitope model

## Abstract

This study examined the genetic and evolutionary features of influenza A/H3N2 viruses in Hangzhou (2010–2022) by analyzing 28,651 influenza-like illness samples from two sentinel hospitals. Influenza A/H3N2 coexisted with other subtypes, dominating seasonal peaks (notably summer). Whole-genome sequencing of 367 strains was performed on GridION platforms. Phylogenetic analysis showed they fell into 16 genetic groups, with multiple clades circulating simultaneously. Shannon entropy indicated HA, NA, and NS gene segments exhibited significantly higher variability than other genomic segments, with HA glycoprotein mutations concentrated in antigenic epitopes A–E. Antiviral resistance showed no inhibitor resistance mutations in PA, PB1, or PB2, but NA mutations were detected in some strains, and most strains harbored M2 mutations. A Bayesian molecular clock showed the HA segment exhibited the highest nucleotide substitution rate (3.96 × 10^−3^ substitutions/site/year), followed by NA (3.77 × 10^−3^) and NS (3.65 × 10^−3^). Selective pressure showed A/H3N2 strains were predominantly under purifying selection, with only sporadic positive selection at specific sites. The Pepitope model demonstrated that antigenic epitope mismatches between circulating H3N2 variants and vaccine strains led to a significant decline in influenza vaccine effectiveness (VE), particularly in 2022. Overall, the study underscores the complex circulation patterns of influenza in Hangzhou and the global importance of timely vaccine strain updates.

## 1. Introduction

Influenza A virus remains a significant global health concern, contributing to an estimated annual mortality of 300,000–650,000 individuals worldwide [1]. Among its subtypes, the H3N2 strain represents a major public health challenge due to its capacity to induce respiratory infections through continuous antigenic evolution [2]. This virus is classified alongside A(H1N1) pdm09, B-Yamagata, and B-Victoria lineages as principal components of seasonal influenza epidemics. These four strains form the basis of the WHO-recommended quadrivalent vaccine formulation, as determined by the WHO Collaborating Centers for Reference and Research on Influenza (available at: https://www.who.int/initiatives/global-influenza-surveillance-and-response-system, accessed on 15 December 2024).

The annual influenza vaccine is an important tool for preventing influenza-like illness (ILI) and reducing the risk of severe complications, particularly among high-risk populations such as young children, older adults, and individuals with chronic medical conditions [3]. Compared to other subtypes, the influenza A(H3N2) strain evolves more rapidly and has spread extensively across multiple countries since its emergence in 1968 [4,5]. Due to the rapid mutation rate of the A(H3N2) virus, new strains may emerge that are not targeted by the annual vaccine. Hangzhou is an international business and tourism city with a total population of 17 million and located along the southeast coast of China with a humid, subtropical climate facilitating the airborne influenza virus survival, transmission, and incidence. As an international transportation hub, Hangzhou exhibits heightened vulnerability to the importation of diverse H3N2 viral variants. Phylogenetic analyses indicate that locally circulating H3N2 strains frequently incorporate exogenous genetic lineages, which necessitates vaccine strain selection strategies to account for multiregional epidemic profiles. However, the WHO-recommended global vaccine strains often fail to promptly address locally emerging antigenic variants due to temporal delays in strain updating and regional surveillance gaps [6]. These challenges highlight the importance of implementing continuous surveillance programs in human populations to monitor circulating strains.

In the post-pandemic of A(H1N1) pdm09, A(H3N2) viruses commenced and predominated in Hangzhou from August 2010 and have become the most important seasonal influenza viruses, causing 16 epidemics in the locality for 13 consecutive years. Our prior studies on H3N2 influenza evolution have primarily utilized HA/NA-targeted Sanger sequencing, revealing antigenic drift patterns in HA1 domains associated with immune evasion [7,8]. However, this gene-specific approach restricts comprehensive understanding of genomic evolutionary dynamics due to technical constraints. We hypothesize that sustained transmission pressure accelerates H3N2 evolution in Hangzhou, driving antigenic drift rates above global averages, while imported lineages shape local viral diversity, yielding antigenic divergence from WHO vaccine strains. To our knowledge, this is the first comprehensive and systematic analysis through 13-year (2010–2022) whole-genome sequencing surveillance of A(H3N2) viruses in Hangzhou, integrating multidimensional evolutionary analysis to establish critical public health resources. Our findings demonstrate accelerated viral evolution in H3N2 lineages and antigenic mismatch between H3N2 variants and vaccine strains reduced influenza vaccine effectiveness (VE), underscoring the critical importance of sustained genomic surveillance to inform both vaccine strain selection and therapeutic countermeasure development.

## 2. Materials and Methods

### 2.1. Study Population and Specimen Collection

Since January 2010, influenza surveillance has been consistently conducted for 13 years in Hangzhou among patients with influenza-like illness (ILI) admitted to two tertiary hospitals, which serve as sentinel sites for an early warning system for pandemic influenza. Clinical information collected included the patient’s name, age, gender, address, symptoms, and date of onset. In total, 28,651 nasopharyngeal/oropharyngeal (NP/OP) swabs and/or tracheal aspirate samples were collected in viral transport medium (VTM) and sent to the Hangzhou Center for Disease Control and Prevention for influenza virus screening within 24 h.

### 2.2. Virus Detection and Subtype Identification

Viral RNA was extracted from respiratory specimens with the RNeasy Mini Kit (Qiagen, Hilden, Germany) according to the standardized protocol. The extracted RNA underwent molecular screening to detect influenza A and B viruses (IAV/IBV) through reverse transcription PCR. IAV-positive specimens were subsequently subjected to subtype discrimination between A(H1N1) pdm09 and A(H3N2) viruses using a multiplex real-time RT-PCR assay kit (Beijing Applied Biological Technologies, Beijing, China) optimized for simultaneous subtype identification.

### 2.3. Genome Sequencing and Assembly

A total of 367 A(H3N2) virus-positive specimens meeting the RT-PCR cycle threshold (Ct) criterion of <28.0 (indicative of high viral load) were selected for genomic sequencing analysis. Selection ensured proportional representation across two surveillance centers and seasonal distribution (summer/winter periods). Viral RNA was used as a template to amplify and acquire a complete viral genome. The multisegmented genome was captured using the ABT Influenza A Virus Genome Capture Kit. Amplification products underwent purification with AMPure XP magnetic beads (Beckman Coulter, Brea, CA, USA; Cat# A63881) prior to library preparation using the Oxford Nanopore Technologies (ONT) SQK-LSK109 workflow. DNA integrity was optimized through sequential treatment with NEBNext FFPE Repair Mix (New England Biolabs, Ipswich, MA, USA; Cat# M6630) and NEBNext Ultra II End Repair/dA-tailing Module (New England Biolabs, Ipswich, MA, USA; Cat# E7546). Barcode integration and adapter ligation were performed using SQK-NBD114.96 and EXP-NBD196 multiplexing kits (Oxford Nanopore Technologies, Oxford, UK). Quantification of nucleic acid samples was conducted using the Qubit 3.0 Fluorometer (Invitrogen Invitrogen, Waltham, MA, USA) with the dsDNA HS Assay System (Thermo Fisher Scientific, Waltham, MA, USA; Cat# Q33231). Prepared libraries were loaded onto FLO-MIN106D flow cells (R9) for sequencing on the GridION platform. Post-sequencing data processing involved quality filtering with Filtlong v0.2.0 (retain reads ≥ 300 bp and Phred score ≥ 10) and genome assembly using Minimap2 (v2.24).

### 2.4. Phylogenetic Analysis, Estimation of Evolution Rate, and Positive Selection Analysis

For comparative analysis, nucleotide sequences of eight amplified genomic fragments obtained in this study were integrated with publicly available reference sequences of A(H3N2) strains retrieved from the Global Initiative on Sharing Avian Influenza Data (GISAID) repository (Table S1). Multiple sequence alignment was performed using MUSCLE v3.8.425 under default parameters. Maximum likelihood phylogenetic reconstruction for the HA gene was implemented in MEGA v11.0.11 with the following specifications: Hasegawa–Kishino–Yano (HKY) substitution model with gamma-distributed rate variation validated through jModelTest, supported by 1000 bootstrap replicates. Phylogenetic visualization was conducted using the iTOL platform (https://itol.embl.de, accessed on 20 December 2024). Evolutionary rate estimation for all eight genomic segments was performed through Bayesian Markov chain Monte Carlo (MCMC) analysis in BEAST v2.7.3 [9]. The analysis employed an uncorrelated lognormal relaxed molecular clock model combined with a coalescent Bayesian skyline tree prior [10]. For nucleotide substitution modeling, we adopted the HKY + gamma model. The MCMC chains were executed for 100 million generations, with parameter sampling conducted at 10,000-step intervals. Convergence of the MCMC runs was verified using Tracer v1.7.2, ensuring all parameters achieved effective sample sizes (ESSs) exceeding the threshold of 200. To quantify amino acid variability, Shannon entropy profiles were generated for each alignment position using the bio3d v2.4.4 package [11,12]. Selection pressure analysis was conducted through three complementary approaches: single-likelihood ancestor counting (SLAC), mixed effects model of evolution (MEME), and fixed effects likelihood (FEL) methods [13,14,15]. These computational analyses were executed through the Datamonkey web interface (http://www.datamonkey.org, accessed on 12 December 2024) of the HyPhy v2.5.67 evolutionary framework, focusing on the identification of codon positions under positive selection pressure.

### 2.5. Vaccine Efficacy and Antiviral Resistance

To estimate the potential vaccine efficacy (VE), the Pepitope measure of antigenic distance established previously was used to calculate the value of VE, where VE = −2.471 × Pepitope + 0.468 [16,17]. A negative value of VE suggests a suboptimal vaccine efficacy against the circulating strains. To express vaccine efficacy (VE) relative to the baseline value (where 0.47 corresponds to 100%), the formula is normalized as follows:

$$VE=\left(\frac{-2.47\times Pepitope+0.47}{0.47}\right)\times 100\%$$

An assessment of the amino acid sequences for all PA, PB1, PB2, NA, and M2 in the isolated A/H3N2 strain was performed to identify the critical mutations known to provide resistance to inhibitors using FluSurver (https://flusurver.bii.a-star.edu.sg/, accessed on 20 December 2022).

## 3. Results

### 3.1. The Prevalence of Influenza A(H3N2) Viruses in Hangzhou from 2010 to 2022

**Figure 1:**
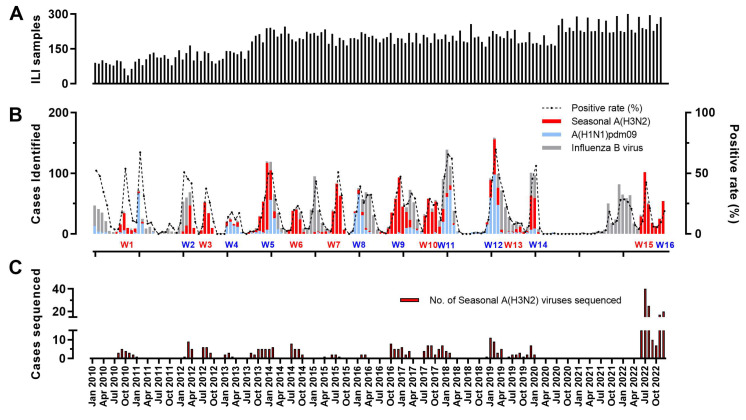
The prevalence of influenza-like illness (ILI) cases at two sentinel hospitals in Hangzhou, China, during the 2010–2022 seasons. (**A**) Distribution of influenza-like illness cases sampled monthly during a 13-year surveillance period. (**B**) The positivity rate of the currently circulating subtypes of human seasonal influenza among influenza-like cases; sixteen wave influenza virus epidemics are seen (W1, 2010–2011 summer; W2, 2011–2012 winter; W3, 2012–2013 summer; W4, 2012–2013 winter; W5, 2013–2014 winter; W6, 2014–2015 summer; W7, 2015–2016 summer; W8, 2015–2016 winter; W9, 2016–2017 winter; W10, 2017–2018 summer; W11, 2017–2018 winter; W12, 2018–2019 winter; W13, 2019–2020 summer; W14, 2019–2020 winter; W15, 2022–2023 summer; W16, 2022–2023 winter). Blue font, winter influenza peaks; red font, summer influenza peaks. (**C**) The sequencing number of A(H3N2) virus-positive samples between 2010 and 2022.

The Hangzhou Influenzas surveillance network was part of the National Influenzas Surveillance Network of China. From January 2010 to December 2022, a total of 28,651 influenza-like illness (ILI) specimens in the two sentinel hospitals were collected and subjected to influenza virus nucleic acid testing (Table S2 and Figure 1A). Among them, 4621 cases tested positive for influenza virus, with a total positivity rate of 16.13%. Influenza viruses were frequently detected in ILI specimens between 2010 and 2022, with observed positivity rates reaching 50–70% in certain months (e.g., February 2019) (Figure 1B). These proportions suggest that influenza may contribute substantially to seasonal ILI burden in the study period, while no positive cases of influenza virus were detected in the 2020–2021 monitoring year (Figure 1B). Moreover, influenza virus (sub)typing results demonstrated the subtypes mainly include A(H1N1) pdm09 (3.11%), A(H3N2) (6.66%), and influenza B virus (6.36%) and alternating dominance by different subtypes over the epidemic seasons (Figure 1B). Notably, among influenza (sub)types, A/H3N2 circulated in Hangzhou throughout the surveillance period, and it dominated all summer influenza peaks after 2010 except for the winter of 2016–2017 (Table S2 and Figure 1B). In total, 367 (19.23%) A(H3N2) virus-positive samples were selected for the whole-genome sequencing analysis, and the sampling distribution and sequencing sample number per quarter are displayed in Figure 1C and Table S2 (Figure 1C).

### 3.2. Phylogenetic Analysis and Clade Identification of Influenza A(H3N2) Viruses

**Figure 2:**
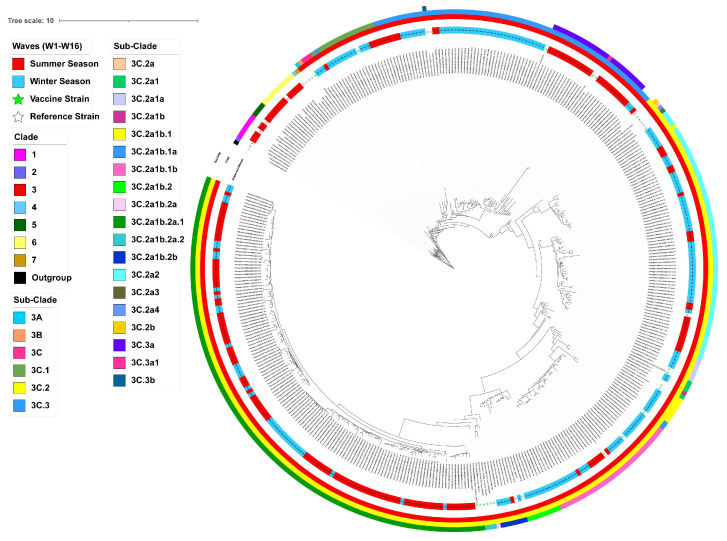
The phylogenetic tree of the HA gene of influenza A(H3N2) virus from local circulating influenza A(H3N2) strains in Hangzhou between 2010 and 2022. The WHO-recommended vaccine strains of the northern hemisphere and reference clades from GISAID were included and annotated in the trees. The tree was constructed by the maximum likelihood method using MEGA v11.0.11 and visualized using the iTOL. Maximum likelihood phylogenies were estimated by the Hasegawa–Kishino–Yano model (HKY) and gamma distribution with bootstrap analysis (1000 replicates). The tree was rooted with A/Victoria/208/2009 as an outgroup.

Pairwise analysis comparing the hemagglutinin (HA) protein amino acid sequences of Hangzhou A/H3N2 viruses with those of contemporaneous vaccine strains revealed amino acid identity levels ranging from 95.94% to 99.47% (Table S2). This highlights genetic variability between circulating strains and vaccine formulations over the study period. Phylogenetic analysis revealed that seasonal influenza A/H3N2 viruses belonged to sixteen genetic groups, including 1, 5, 6, 3C, 3C.1, 3C.3, 3C.3a, 3C.2a1a, 3C.2a1, 3C.2a2, 3C.2a1b.1, 3C.2a1b.1b, 3C.2a1b.2, 3C.2a1b.2b, 3C.2a1b.1a, and 3C.2a1b.2a.1 (Figure 2 and Table S2). Influenza A/H3N2 isolates from W1 (2010–2011 summer) predominantly belonged to clades 1, 5, and 6. Most isolates from W2 (2011–2012 winter) and some isolates from W3 (2012–2013 summer) clustered within subclade 3C.1. The subclade 3C.3 contained A/H3N2 strains from W3, W4 (2012–2013 winter), and W5 (2013–2014 winter), while W6 (2014–2015 summer) and the majority of W7 strains (2015–2016 summer) belonged to the subclade 3C.3a. We found that all strains circulating in W8 (2015–2016 winter) and some in W9 (2016–2017 winter) belonged to subclade 3C.2a1a. Subclade 3C.2a2 continuously dominated in W9, W10 (2017–2018 summer), and W11 (2017–2018 winter). In addition, the A/H3N2 viruses circulating in W12 (2018–2019 winter) diverged into four subclades, including subclade 3C.2a1b.1b, 3C.2a1b.2, 3C.2a1b.2b, and 3C.2a1b.1. Among which, subclade 3C.2a1b.1b was dominant in strains circulating in W12, W13 (2019–2020 summer), and W14 (2019–2020 winter). All A/H3N2 strains circulating in W15 (2022–2023 summer) and W16 (2022–2023 winter) belonged to the subclade 3C.2a1b.2a.1.

### 3.3. Shannon Entropy Diversity

**Figure 3:**
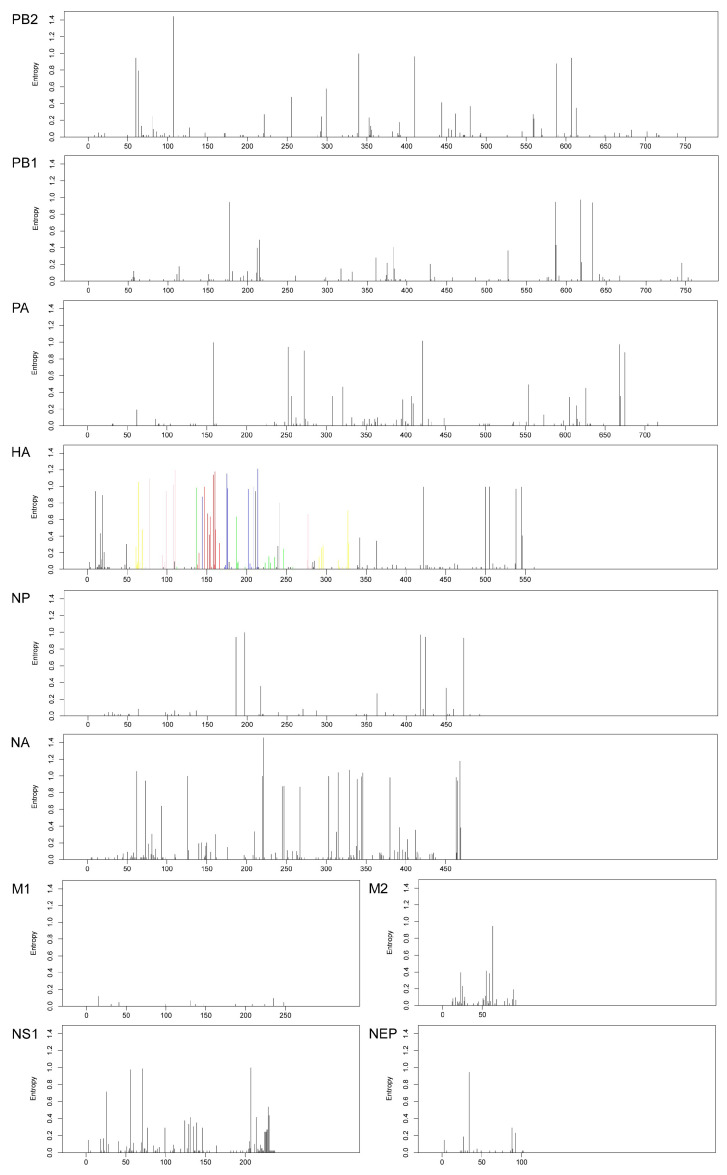
Shannon entropy for each genome segment of influenza A/H3N2 strains in Hangzhou, 2010–2022. Each bar represents the Shannon entropy value of each amino acid position in each genome segment. The Shannon entropy value of positions in known antigenic epitope regions A (red; amino acid residues 122, 124, 126, 130–133, 135, 137, 138, 140, 142–146, 150, 152, 168), B (blue; residues 128, 129, 155–160, 163, 165, 186–190, 192–194, 196–198), C (yellow; residues 44–48, 50, 51, 53, 54, 273, 275, 276, 278–280, 294, 297, 299, 300, 304, 305, 307–312), D (green; residues 96, 102, 103, 117, 121, 167, 170–177, 179, 182, 201, 203, 207–209, 212–219, 226–230, 238, 240, 242, 244, 246–248), and E (pink; residues 57, 59, 62, 63, 67, 75, 78, 80–83, 86–88, 91, 92, 94, 109, 260–262, 265) of HA protein in the dominant circulating strains were labeled. The amino acid numbering is counted without the signal peptide.

Shannon entropy, serving as a robust metric for evaluating residue variability at individual alignment positions, demonstrates that elevated entropy values correlate with increased amino acid substitution propensity at corresponding sites [11,12]. Quantitative analysis of Shannon entropy revealed distinct evolutionary dynamics among H3N2 genomic segments in the studied population. The HA, NA, and NS gene segments exhibited consistently higher entropy values relative to PA, PB1, PB2, M1, M2, and NEP, indicative of heightened genetic diversification within these segments (Figure 3).

Structural dissection of the HA1 domain identified six distinct regions, comprising canonical antigenic epitopes A–E and non-epitope-associated positions. Notably, epitopes A–E exhibited higher sequence diversity compared to non-epitope regions, a pattern likely driven by immune-driven selection pressures, given their role as primary targets for neutralizing antibodies. A total of 127 amino acid substitutions were identified in the neuraminidase (NA) protein, with the majority occurring outside framework regions and catalytic residue positions. Among the clinical isolates analyzed, 96.2% (353/367) remained sensitive to neuraminidase inhibitors (NAIs). However, resistance-associated mutations were detected in the remaining 3.8% (14/367) of samples, including I222V substitution (one isolate), Y155H substitution (one isolate), Y155F + D251V dual mutations (three isolates), D251N substitution (two isolates), V149I/L substitutions (seven isolates) (Table S3), which highlight the need for ongoing surveillance of key resistance-associated residues (e.g., Y155, V149, I222, and D251) to monitor potential shifts in antiviral susceptibility. Sequence analysis identified 75 amino acid variations in the NS1 protein, with elevated entropy value localized to the effector domain and C-terminal tail. Notably, 14 strains harbored an R224X termination mutation (arginine-to-stop-codon substitution), while one strain exhibited a Q218X mutation (glutamine-to-stop-codon), collectively abolishing the PDZ-binding motif critical for host–pathogen interactions (Table S4). Furthermore, a novel seven-residue C-terminal extension was detected in the NS1 protein of the A/Hangzhou/19H31101A/2019 isolate (Table S4), suggesting potential structural or functional divergence in recent variants. M2 protein analysis showed that 67 amino acid positions underwent variations and 99.72% (366/367) of isolates carried the S31N mutation, a primary marker of adamantane resistance. Secondary mutations were identified in specific strains: V27I in the 2012 isolate (A/Hangzhou/12H30763CS/2012) and V27A in the 2013 isolate (A/Hangzhou/13H30536A/2013).

### 3.4. Evolutionary Rates and Selection Pressure Analysis

Detailed evolutionary rates and selection pressure analysis for all segments of the A(H3N2) genome are shown in Table 1. The average nucleotide substitution rates of the individual segments varied with the highest rates occurring in the key glycoproteins of HA (3.96 × 10^−3^ substitutions per site per year), followed by the NA segment (3.77 × 10^−3^ substitutions per site per year) and NS (3.65 × 10^−3^ substitutions per site per year). The lowest evolutionary rates were noted for the NP segment (2.62 × 10^−3^ substitutions per site per year). The SLAC approach was applied to calculate the dN/dS ratio (non-synonymous-to-synonymous substitution ratio) across all eight viral genomic segments, providing insights into evolutionary selective pressures. The analysis revealed that NS1 and M2 proteins displayed the most rapid evolution, with dN/dS values of 0.346 and 0.464, respectively. HA (0.218), NA (0.266), and NEP (0.205) exhibited moderate evolutionary rates, and PB1, PB2, PA, M1, and NP proteins showed significantly lower dN/dS ratios (ranging from 0.037 to 0.096). Three robust computational methods (SLAC, MEME, and FUBAR) were employed to identify sites under positive selection across all eight influenza genomic segments. Selective pressure analysis results showed that no positively selected sites were detected in the NEP, PB1, or M1 segments across all models. In contrast, two positively selected sites within the HA segment (AA at 21 and 176), two in the NA segment (AA at 93 and 380), and one in the PB2 segment (AA at 67) were all detected across all three models. Additionally, the PA segment (AA at 321) and M2 segment (AA at 28 and 68) showed one and two common diversifying positive selections, respectively, in all MEME and FUBAR models (Table 1). These results highlight distinct evolutionary pressures across viral proteins, with HA, NA, PB2, PA, and M2 exhibiting signatures of adaptive evolution, while NEP, PB1, and M1 remain under strong functional constraints.

### 3.5. Antigenic Characteristics and Vaccine Strain Match Analysis

**Figure 4:**
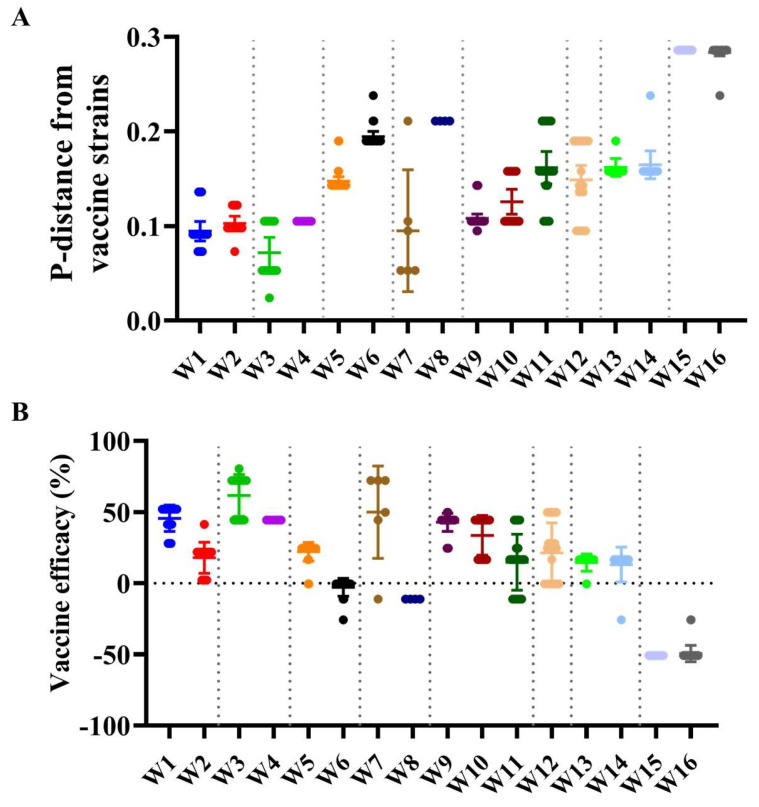
P-distance and prediction of vaccine efficacy against influenza A/H3N2 strains in Hangzhou, 2010–2022. Annual vaccine candidates from corresponding epidemics are separated by a dotted line. (**A**) The P-distance values of the HA1 substitutions on epitope responsible for vaccine mismatch. (**B**) Prediction of potential vaccine efficacy (VE) against the circulating influenza A/H3N2 strains.

We next used the Pepitope model, which considers the distinct antigenic sites between circulating strains and the vaccine virus by considering the epitope sites to evaluate how closely the vaccine strain resembles the circulating strains in Hangzhou (Figure 2 and Figure 4A,B and Table S5). For W1 and W2 influenza seasons, the antigenic distance of the HA1 gene between the vaccine strain A/Perth/16/2009 and the Hangzhou isolates was 0.095 (dominant epitope = E; mutations: K62E, N81D, K92E, Y94H, I260M, R261Q) and 0.102 (dominant epitope = C; mutations: S45N, S47T, T48I, D53N, N278K, E280A, N278K, N312S), suggesting a vaccine efficacy (VE) of 45.63% and 17.87%. For the W3 and W4 seasons, the antigenic distance between A/Victoria/361/2011 and the Hangzhou isolates was 0.072 (dominant epitope = A; mutations: S124N, R142G, N145S) and 0.105 (dominant epitope = A; mutations: A138S, R142G, N145S), with vaccine efficacy (VE) of 61.64% and 44.42%. For W5 and W6 seasons, the distance between A/Texas/50/2012 and the Hangzhou isolates was 0.150 (dominant epitope = B; mutations: N128A, L157S, V186G, P198S) and 0.195 (dominant epitope = B; mutations: N128A, F159S, V186G, F193S, P198S), with vaccine efficacy (VE) of 22.18% and -2.89%. For W7 and W8 seasons, the distance between A/Switzerland/9715293/2013 and the circulating strain was 0.095 (dominant epitope = A; mutations: S124I, S138A, R140I, G142R, N144S) and 0.211 (dominant epitope = A; mutations: S138A, R140I, G142R, N144S, with vaccine efficacy (VE) of 49.94% and −11.16%. For W9, W10, and W11 seasons, the distances between A/Hong_Kong/4801/2014 and the circulating strain were 0.108 (dominant epitope = A; mutations: T131K, R142K/G, R150K), 0.126 (dominant epitope = A; mutations: S124G, T131K, R142K, T135K, S144K, R150K), and 0.161 (dominant epitope = A; mutations: T131K, R142K/G, T135K, S144K, R150K), with vaccine efficacy (VE) of 42.79%, 33.61%, and 14.74%. For W12 seasons, the distance between A/Singapore/INFIMH-16-0019/2016 and the circulating strain was 0.149 (dominant epitope = B; mutations: T128A, K160T, F193S, P194L, Q197R), with vaccine efficacy (VE) of 21.30%. For W13 and W14 seasons, the distance between A/Kansas/14/2017 and the circulating strain was 0.162 (dominant epitope = A; mutations: T135K, S137F, K144S) and 0.165 (dominant epitope = A; mutations: T135K, S137F, K144S), with vaccine efficacy (VE) of 14.48% and 13.10%. For W15 and W16 seasons, the distance between A/Darwin/6/2021 and the circulating strain was 0.286 (dominant epitope = B; mutations: S156H, N159Y, I160T, D186S, N190D, S198P), with vaccine efficacy (VE) of −50.85% and−49.49%.

## 4. Discussion

Influenza epidemics exhibit distinct seasonal patterns globally [4,18]. Scientific studies have demonstrated an epidemiological association between influenza A(H3N2) and summer epidemics, underscoring the importance of seasonal monitoring for public health [19,20]. Therefore, this study systematically assessed the epidemiological impact of influenza types (A/B) and subtypes (pH1N1/H3N2) on outbreak dynamics, while concurrently analyzing seasonal transmission patterns across Hangzhou, China, over 13 years (2010–2022), to characterize region-specific viral behavior. This investigation revealed a marked predominance of A/H3N2 in summer influenza cases, while winter and spring exhibited a dynamic interplay between circulating subtypes (pH1N1, H3N2, and influenza B viruses) (Figure 1B), underscoring the region’s complex epidemiological profile and the necessity for subtype-specific surveillance strategies. At the end of 2019, severe acute respiratory syndrome coronavirus 2 (SARS-CoV-2) emerged, rapidly spread worldwide, and caused the COVID-19 pandemic. Mirroring national public health strategies, Hangzhou maintained stringent non-pharmaceutical interventions (NPIs) until late 2022 [21,22]. These measures, targeting respiratory transmission pathways shared by SARS-CoV-2 and influenza viruses, substantially disrupted local influenza circulation from 2020–2021. Notably, the 2022 relaxation of COVID-19 regulations with the global easing of containment policies triggered a resurgence of influenza activity in Hangzhou, dominated by the B/Victoria lineage and A(H3N2) subtype (Figure 1B), highlighting the interplay between pandemic policies and seasonal respiratory pathogen dynamics.

Comparative analysis across the eight genomic segments revealed that the HA gene exhibited the highest evolutionary rate, significantly outpacing other viral segments (Figure 3 and Table 1). The calculated evolutionary rate of the HA gene in this study aligns with the estimates observed in other regions (3.37 × 10^−3^–4.84 × 10^−3^) [10,23]. However, this contrasts with findings from subtropical regions such as Bhutan and Bangladesh, where the non-structural (NS) gene exhibits the highest evolutionary rates [5], potentially reflecting distinct host–pathogen adaptation mechanisms or environmental drivers in these ecosystems. In addition, accelerated genomic evolution was similarly observed in the NS, NA, and HA gene segments (Table 1), as evidenced by their elevated overall dN/dS ratios, a pattern consistent with findings from prior studies [24]. The selection pressure analysis demonstrated that Hangzhou A(H3N2) strains were overwhelmingly dominated by purifying selection, with only sporadic positive selection signals detected at specific sites (Table 1). This suggests that adaptive mutations occur episodically, while stochastic genetic drift predominantly governs the local evolutionary dynamics of influenza A viruses, with minimal contribution from sustained directional selection.

Antiviral target analysis revealed that, while PA, PB1, PB2, NA, and M2 proteins are critical for influenza antiviral resistance development [25], no resistance-associated mutations were detected in the inhibitor-binding sites of PA, PB1, or PB2 in the studied strains (Figure 3). This absence of genetic alterations in resistance confirms the retained therapeutic efficacy of corresponding inhibitors against these Hangzhou influenza viruses, highlighting their current clinical utility. The NA protein promotes the release of viral particles from host cells via enzymatic activity, which consists of eight highly conserved residue active sites (R118, D151, R152, R224, E276, R292, R371, and Y406) and an outer shell of 10 framework residues (E119, R156, W178, S179, D198, I222, E227, E277, N294, and E425) [26]. Although 96.2% of Hangzhou A(H3N2) viruses remained sensitive to neuraminidase inhibitors (NAIs), mutations associated with resistance to oseltamivir and zanamivir, such as V149I/L, I222V, Y155H, Y155F + D251V, and D251N, were detected in NA protein in some strains. These site-specific alterations may differentially impact viral replicative capacity, transmissibility, and drug susceptibility by modulating enzymatic activity or inhibitor binding efficiency [26]. A single mutation in the M2 protein transmembrane domain (positions 26, 27, 30, 31, or 34) can confer adamantane resistance [4]. In line with global trends [25], 99.72% (366/367) of Hangzhou A(H3N2) strains harbored the dominant resistance marker S31N, with additional secondary mutations (V27I and V27A) observed in subsets of viruses. These findings reinforce adamantane’s limited clinical utility against circulating strains and emphasize the imperative for sustained antiviral resistance surveillance to guide therapeutic strategies.

NS1, influenza A virus non-structural protein, is critical for viral replication and pathogenesis. The NS1 protein plays a crucial role in the evolution of viruses and their ability to transmit between different species, which comprises an N-terminal dsRNA-binding domain (residues 1–73), linker region (residues 74–84), effector domain (residues 85–201), and C-terminal tail (residues 202–230) [27]. The terminal four residues at the C-terminus of the NS1 protein harbor a consensus motif for PDZ-domain ligands, facilitating binding to proteins with PDZ domains, which is vital for the virus’s replication and pathogenicity [27]. Genomic analysis of Hangzhou A(H3N2) strains identified 14 variants harboring premature termination mutations in the NS1 protein, resulting in a complete loss of PDZ-binding motifs (Figure 3). This observation aligns with prior reports of NS1 truncations in Jining City, from 2018 to 2020 [28], yet the scale of truncation events (14 R224X variants) within a single geographic cohort is unprecedented. The heightened prevalence of NS1 truncations in Hangzhou A(H3N2) strains warrants investigation into host-mediated selective pressures, including potential drivers such as population immunity dynamics, antiviral-induced mutagenesis, or coinfection-driven adaptation.

Long-term epidemiological surveillance reveals frequent antigenic mismatch events, with vaccine effectiveness (VE) significantly declining when vaccine strains fail to match circulating variants [6]. Following the 2009 H1N1 pandemic, H3N2 viruses exhibited increased genetic complexity, with multiple clades cocirculating simultaneously (Figure 2, Table S2). Notably, paradoxical negative VE against A(H3N2) was observed during the 2022 season in China, aligning with reports from the US [29], Italy [30], and Senegal [4], collectively suggesting suboptimal protection across diverse geographic and demographic populations. In addition, this unprecedented phenomenon likely arose from prolonged COVID-19 NPIs suppressing influenza transmission, creating an “immune debt” that accelerated viral diversification. By 2022, critical antigenic B-site mutations (e.g., S156H, N159Y, I160T, D186S, N190D, S198P) in dominant clade 3C.2a1b.2a.1 strains substantially diverged from the A/Darwin/6/2021 vaccine strain. These findings highlight NPIs’ dual impact: while curbing transmission, prolonged use may inadvertently drive viral evolution and reduce VE. The global emergence of negative VE underscores the urgency for adaptive strategies, including real-time antigenic monitoring and universal vaccine development.

## 5. Limitations and Strengths

Our study has some limitations. First, the 13-year surveillance data from two urban hospitals in Hangzhou may limit generalizability to broader regions. Second, key genomic observations (NS1 truncations, HA substitutions) require experimental validation through neutralization assays or reverse genetics to confirm biological impacts on viral fitness. Third, the failure to effectively integrate sequencing data with clinical data represents a critical knowledge gap. Nevertheless, this work also establishes significant advances: (1) the first 13-year whole-genome sequencing resource for A(H3N2) in subtropical China (Hangzhou), comprising 367 genomes across 16 epidemic waves, provides critical insights into vaccine effectiveness (VE); (2) comprehensive evolutionary analysis integrating phylogenetic, entropy, molecular clock, and selection pressure methods across all viral segments, overcoming previous HA/NA-centric biases in our studies.

## Figures and Tables

**Table 1:** The estimated evolutionary rate and selection pressure analysis for all segments of A(H3N2) viruses circulating in Hangzhou between 2010 and 2022.

Segments (*n* = 367)	Evolutionary Rate (95% HPD)	Protein	dN/dS	Sites Under Diversifying Positive Selection (Amino Acid Position)		
				SLAC ^a^	MEME ^a^	FUBAR ^b^
HA	3.96 × 10^−3^ (3.53–4.38)	HA	0.218	21, 176, 277	21, 26, 53, 110, 147, 176, 209	21, 69, 110, 176, 209, 277
NA	3.77 × 10^−3^ (3.31–4.24)	NA	0.266	93, 380	45, 93, 370, 380	93, 380
NS	3.65 × 10^−3^ (2.92–4.42)	NS1	0.346	None	None	22, 71, 230
		NEP	0.205	None	None	None
PB1	3.09 × 10^−3^ (2.67–3.57)	PB1	0.072	None	None	None
PB2	2.91 × 10^−3^ (2.50–3.30)	PB2	0.076	67	67, 513	67
PA	2.81 × 10^−3^ (2.47–3.16)	PA	0.094	None	32, 109, 321, 500, 628	272, 321
MP	2.63 × 10^−3^ (2.16–3.10)	M1	0.037	None	None	None
		M2	0.464	None	28, 68	28, 59, 68
NP	2.62 × 10^−3^ (2.21–3.06)	NP	0.060	None	None	270

^a^ *p* value threshold of 0.1. ^b^ Posterior probabilities of 0.9.

## Data Availability

The original contributions presented in this study are included in the article/Supplementary Material. Further inquiries can be directed to the corresponding author(s).

## Supplementary Materials

The following supporting information can be downloaded at: https://www.mdpi.com/article/10.3390/v17040526/s1, Table S1: Origin of Genome sequence information of reference A(H3N2) strains included in the phylogenetic analyses; Table S2: The clade of WHO-recommended H3N2 vaccine strains and concurrent most dominant strains in Hangzhou; Table S3: Mutations associated with resistance to NAI of influenza A/H3N2 strains in Hangzhou, from 2010 to 2022; Table S4: Mutations associated with loss of PDZ-binding motifs in NS1 protein; Table S5: Efficacy estimation among influenza A/H3N2 vaccine strains and mutations found on the dominant epitopes of influenza A/H3N2 strains in Hangzhou, from 2010 to 2022.

## Abbreviations

HPD	Highest probability density
dN/dS	Non-synonymous/synonymous rate ratio
SLAC	Single-likelihood ancestor counting
MEME	Mixed effects model of evolution
FUBAR	Fast, unconstrained Bayesian approximation
